# Dietary Carbohydrate and Nocturnal Sleep Duration in Relation to Children’s BMI: Findings from the IDEFICS Study in Eight European Countries

**DOI:** 10.3390/nu7125529

**Published:** 2015-12-08

**Authors:** Monica Hunsberger, Kirsten Mehlig, Claudia Börnhorst, Antje Hebestreit, Luis Moreno, Toomas Veidebaum, Yiannis Kourides, Alfonso Siani, Dénes Molnar, Isabelle Sioen, Lauren Lissner

**Affiliations:** 1 Section for Epidemiology and Social Medicine, University of Gothenburg, P.O. Box 453, 40530 Gothenburg, Sweden; kirsten.mehlig@gu.se (K.M.); lauren.lissner@gu.se (L.L.); 2 Leibniz Institute for Prevention Research and Epidemiology-BIPS GmbH, Achterstrasse 30, D-28359 Bremen, Germany; boern@bips.uni-bremen.de (C.B.); hebestr@bips.uni-bremen.de (A.H.); 3 Growth, Exercise, Nutrition, and Development (GENUD) research group, University of Zaragoza, Domingo Miral, 50009 Zaragoza, Spain; lmoreno@unizar.es; 4 National Institute for Health Development, P.O. Box 3012, 10504 Tallinn, Estonia; toomas.veidebaum@tai.ee; 5 Research and Education Institute of Child Health, 138 Limassol Ave, #205, 2015, Strovolos 510903, Cyprus; kourides@cytanet.com.cy; 6 Institute for Food Sciences, Unit of Epidemiology and Population Genetics, National Research Council, Via Roma 64, 83100 Avellino, Italy; asiani@isa.cnr.it; 7 Department of Paediatrics, Medical Faculty, University of Pécs, Jozsef A.u., 7 H-1062 Budapest, Hungary; molnar.denes@pte.hu; 8 Department of Public Health, Ghent University, 4K3, De Pintelaan 185, 9000 Ghent, Belgium; isabelle.sioen@ugent.be; 9 Research Foundation—Flanders, Egmonstraat 5, B-1000 Brussels, Belgium

**Keywords:** proportion carbohydrate intake at main meals, starch, sugar, childhood overweight, nocturnal sleep duration, breakfast consumption

## Abstract

Previous research has found an association between being overweight and short sleep duration. We hypothesized that this association could be modified by a high carbohydrate (HC) diet and that the timing and type (starch or sugar) of intake may be an important factor in this context. Participants in the prospective, eight-country European study IDEFICS were recruited from September 2007 to June 2008, when they were aged two to nine years. Data on lifestyle, dietary intake and anthropometry were collected on two occasions. This study included 5944 children at baseline and 4301 at two-year follow-up. For each meal occasion (morning, midday, and evening), starch in grams and sugar in grams were divided by total energy intake (EI), and quartiles calculated. HC-starch and HC-sugar intake categories were defined as the highest quartile for each meal occasion. In a mutually adjusted linear regression model, short sleep duration as well as HC-starch in the morning were positively associated with body mass index (BMI) z-scores at baseline. HC-starch at midday was positively associated with body mass index (BMI) z-scores in children with short sleep duration, and negatively associated with BMI z-scores in those with normal sleep. After adjustment for baseline BMI z-scores, associations between total HC from starch or sugar and high BMI z-scores at two-year follow-up did not persist. Our observations offer a perspective on optimal timing for macronutrient consumption, which is known to be influenced by circadian rhythms. Reduced carbohydrate intake, especially during morning and midday meals, and following nocturnal sleep duration recommendations are two modifiable factors that may protect children from being overweight in the future.

## 1. Introduction

Childhood obesity is a serious threat to public health due to long-term consequences, including chronic disease in early adulthood [1]. Overweight and obesity are believed to be preventable conditions, although the issue of effective interventions for primary prevention is not well understood [2] and increased knowledge of modifiable risk factors is needed [3].

Previous investigations have shown that reduced sleep duration is associated with overweight in children and adolescents [4,5,6]. Nielsen *et al.* (2011) report on eight prospective cohort studies of children, all of which confirmed a significant inverse association between hours spent sleeping and future weight gain or development of obesity [4]. Findings from a Belgian longitudinal study confirmed that short sleep duration is associated with central adiposity [7]. Additionally, findings from our own cohort, the Identification and prevention of Dietary- and lifestyle-induced health EFfects In Children and infantS (IDEFICS) study, indicated that sleep duration and overweight were associated cross-sectionally, but this association was no longer observed after adjustment for other behavioral factors and for parental education [8].

Carbohydrate, *i.e.*, digestible starch and sugar, is likely to play a significant role in energy balance since it is the major macronutrient impacting blood sugar levels. Low carbohydrate intake is generally defined as <130 g/day or <26% of total energy intake (EI), while 26%–45% of EI from carbohydrates is considered to be moderate carbohydrate intake [9]. A carbohydrate proportion exceeding 45% EI is considered to be moderately high and >60% of EI from carbohydrates is considered to be high, although there is no clear established cut-off [10].

Relatively few studies have investigated the relationship between dietary carbohydrate intake and energy balance in children. However, the Feeding Infants and Toddlers Study (FITS), conducted in the USA, found that toddlers often have low intakes of fruits and green or yellow vegetables, consuming instead a high amount of starchy foods from grains. The authors highlighted this as a potential focus area for early overweight prevention efforts [11]. A study of Malaysian children found that the percentage of total EI represented by carbohydrates was significantly higher in overweight/obese children, compared with normal-weight children [12], suggesting that the proportion of EI from carbohydrates may play a role in childhood obesity.

Breakfast is often high in carbohydrate from both starch and sugar. Furthermore, the relationship between breakfast consumption patterns and overweight is not clear. Eating breakfast has long been portrayed as important, possibly enabling control of overconsumption later in the day. Several studies of breakfast-skipping behavior across European countries have shown that children who eat breakfast have a lower BMI, compared with children who do not [13,14,15,16]. However, a 2010 systematic review reported that breakfast consumption is associated with increased body weight in European children and adolescents in observational studies but that causality was not demonstrated [17]. Brown *et al.* (2013) concurred, stating that the proposed effect of consuming breakfast on obesity is not supported by scientific findings [18].

**Figure 1:**
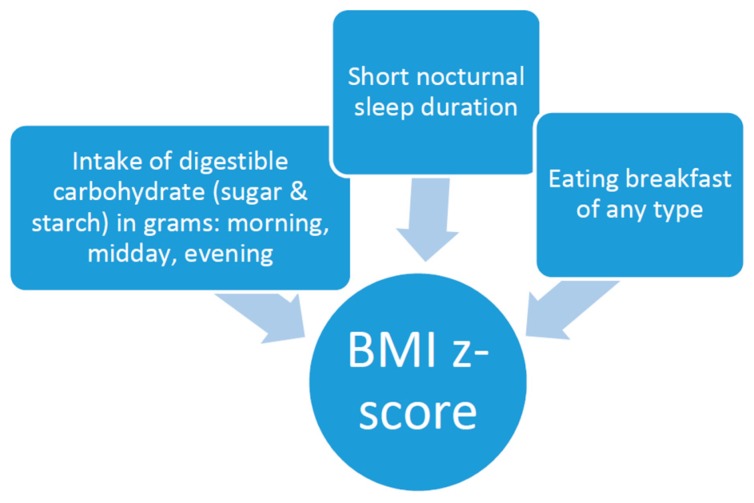
Factors investigated with body mass index (BMI) z-scores. Eating breakfast has a negligible relationship with carbohydrate (sugar and starch) intake during meals. Correlations range from −0.11 (evening sugar) to 0.13 (morning sugar).

Therefore, the aims of this study were to examine whether high intake of carbohydrate modifies the association between short sleep duration and overweight, whether intake of starch and sugar differ in this regard, whether the timing (morning, midday, and evening) of carbohydrate intake is significant, and whether breakfast consumption, regardless of macronutrient composition, minimizes the risk of overweight in a mutually adjusted model (see Figure 1). The study was conducted on a geographically dispersed European sample participating in IDEFICS [19,20].

## 2. Methods

### 2.1. Participants

IDEFICS is a prospective cohort study with an embedded intervention, including eight centers in Europe (Belgium, Cyprus, Estonia, Germany, Hungary, Italy, Spain and Sweden). From September 2007 to June 2008, 16,228 children aged two to nine years underwent the baseline investigation, providing lifestyle and dietary pattern information, anthropometrics and biological samples. Following recruitment, a community intervention with six key health messages was undertaken, including: (1) increasing daily physical activity; (2) decreasing daily screen time; (3) increasing fruit and vegetable intake; (4) drinking more water; (5) getting adequate sleep; and (6) being together with family. From September 2009 to March 2010, the children participated in a follow-up examination.

During the baseline and follow-up examinations, parents or legal guardians provided written informed consent for all examinations and the collection of biological samples, as well for analysis and storage of personal data and collected samples. Survey centers in the eight countries collected data according to standardized operating procedures and adherence to a predefined protocol. All questionnaires were translated from English into the respective national language and then back-translated into English at the respective study centers, in order to ensure accuracy of translations. Detailed information on the study procedures is available in [19,20,21].

### 2.2. Assessment Of Main Exposures: High Carbohydrate Intake and Sleep Duration

Dietary intake was assessed by standardized 24-h dietary recalls (24-h), based on the responses of parents or guardians (hereafter called “parents”) of participating children to the Self -Administered Children and Infant Nutrition Assessment (SACINA). This computer-based instrument, previously created for the Healthy Lifestyle in Europe by Nutrition in Adolescence (HELENA) study, was developed for IDEFICS based on Young Adolescents’ Nutrition Assessment on Computer (YANA-C) software [22,23]. The dietary recall part of the Self Administered Children and Infant Assessment (SACINA) instrument presents, in an interactive menu, country-specific food items with photographs of different portion sizes for the most common items, as well as probing questions regarding usual combinations of foods such as cereal and milk. The information collected through the SACINA was linked to Food Composition Tables (FCT) in order to calculate nutrient intake. The respective national FCT was used in each country except Hungary, where the German FCT was used. Nutrients are presented in standard units, grams and kilocalories (kcal), based on a standard FCT [24].

Data on diet and sleep were collected on all days of the week, including weekends. Most parents completed one 24-h recall for their child, while a subset completed more than one recall. As parents were not able to report on meals consumed at schools or kindergartens, an on-site school meal assessment, using a predefined observation-recording template with standard portions, was carried out by a teacher or school employee. A complete 24-h recall included everything the children ate or drank during their waking hours during one day. The 24-h recall allows for the calculation of energy intake, the proportion of intake from each of the macronutrients, the proportion of carbohydrate kcal from sugar and starch and hours of nocturnal sleep, hereby referred to as sleep. We defined short sleep duration as less than 10 h, based on sleep recommendations for children [25].

We restricted the present sample to interviews recalling intake Monday through Thursday, as a previous investigation on the same sample indicated that dietary intake on Friday lies between weekday and weekend intake [8,26]. Therefore, to minimize heterogeneity we classified Friday as a weekend day and excluded them. In cases with more than one dietary recall, only the first complete weekday record of dietary intake was included in the analysis.

The SACINA instrument is structured around six meals without time parameters: breakfast, mid-morning snack, lunch, afternoon snack, evening meal and evening snack. If an additional snack was indicated in the interview, it was added to a previous snack in order to account for all EI. However, five was the median number of meals consumed and only 20% of children exceeded this number of meals. Eating breakfast was recorded as yes or no, based on the 24-h recall.

Importantly, as under- and over-reporting of dietary intake is a common problem, we examined total daily EI by comparing EI to the basal metabolic rate estimated by the age- and gender-specific Schofield equation [27]. Using the widely-acknowledged Goldberg cut-offs, we classified 254 (3.43%) as over-reporters and 701 (9.45%) as under-reporters and removed these 955 subjects from the analysis [28]. The prevalence of under- and over-reporting is not equally distributed across the countries. Under-reporting ranged from 1.1% in Spain to 18.44% in Cyprus, while over-reporting was most prevalent in Italy (5.6%) and lowest in Sweden (1.1%). When the under- and over-reporting subjects were excluded, Cyprus lost 19.6%, Hungary 19.4%, Germany 14.5%, Belgium 13.0%, Italy 11.5%, Estonia 11.2%, Spain 5.2% and Sweden 4.8%. An additional 107 cases were excluded because macronutrient components did not equal total EI, a result of missing data in the national FCTs.

For each meal occasion (morning, midday, and evening), starch in grams and sugar in grams were divided by total energy intake (e.g., the sum of sugar in grams for breakfast and morning snack/total daily EI). Then, HC intake for starch and sugar on each occasion was defined by assigning those in the highest quartile to the HC-starch and HC-sugar intake categories. Average intakes of starch during morning, midday, and evening meals were 50, 115, and 61 g, respectively. Similarly, average intakes of sugar were 64, 70, and 53 g, for the three meal periods. When describing total carbohydrate intake for the day, percentage of total energy from both carbohydrate sources was used.

### 2.3. Anthropometry and Body Mass Index (BMI)

Anthropometric data were collected at each participating survey center, according to a standard protocol. Body height was measured without shoes by trained research staff using a portable stadiometer (SECA 225). Body weight was measured with an electronic scale (TANITA BC 420 SMA), with subjects wearing light clothing. BMI-z-scores and BMI categories were calculated according to the criteria of the International Obesity Task Force (IOTF) [29]. The same procedure was followed at both examinations (baseline 2007/2008 and follow-up 2009/2010) and inter-observer reliability was assessed at each survey center [30].

### 2.4. Other Factors

Age, sex, highest household parental education level and survey country were included in the multivariable model. Data on parental factors were collected by a standardized parental questionnaire. Age was examined as a continuous variable. Household parental education level was categorized according to the International Standard Classification of Education (ISCED) and the original six ISCED levels were then combined into two levels [31]. ISCED levels 0–4 constitute “not high” education while levels 5 and 6 are defined as high. Moreover, we controlled for intervention when examining BMI z-scores at follow-up, as children in the intervention had been exposed to instructions to get adequate sleep.

**Figure 2:**
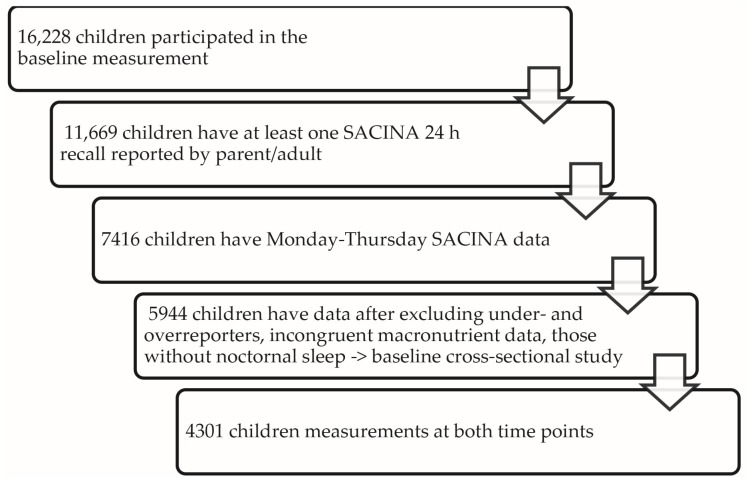
Study participants.

After limiting our sample to the first weekday recall, excluding cases with missing information about school meals or other meals outside of parental control and incomplete recalls, data from 7416 children were available. After excluding over- and under-reporters and children with incomplete sleep data, the baseline sample size was reduced to 5944. The longitudinal sample was further reduced to 4301 as some children were lost to follow-up (see Figure 2).

### 2.5. Statistical Analysis

BMI z-scores and dietary exposures are presented by short sleep duration at baseline. T-tests and regression equations controlling for country and age were used to make comparisons between short sleepers and “not-short” sleepers, presented with group means, regression coefficients and 95% confidence intervals. Linear regression was used for analysis of continuous variables (BMI-z-score, energy kcals/day, starch in grams, sugar in grams and macronutrient proportions) and logistic regression for binary outcomes (high starch and high sugar) at each time-point, eating breakfast (yes or no), high household parental education level); the respective regression coefficients are reported.

At baseline, we used a multivariable regression model to assess the association between BMI z-scores and short sleep duration (<10 h) and high intake of carbohydrate from starch and sugar at three different time-points (morning, midday, evening), while controlling for age, sex and parental education, and including country as a random intercept.

The same baseline variables were included in our prediction of BMI z-scores at two-year follow-up, in which we further controlled for BMI z-scores at baseline and exposure to the IDEFICS intervention, as mentioned above. Cross-sectionally at baseline, we assessed how being in the highest quartile of carbohydrate for starch or sugar modified the association between short sleep duration and BMI z-scores by introducing six product terms, *i.e.*, the dichotomized sleep variable multiplied by high starch and sugar intake at three time-points (morning, midday, evening). We further controlled for age, sex and parental education and included country as a random intercept.

We checked for multicollinearity between predictors and confounders included in the model by estimating the variance inflation factor (VIF), finding that none exceeded 2.0.

The significance level was set at α = 0.05 for all analyses (2-sided tests). Statistical analyses were carried out with Stata Intercool 11.2, StataCorp LP, 4905 Lakeway Drive, College Station, TX, USA.

### 2.6. Ethics Statement

We certify that all applicable institutional and governmental regulations concerning the ethical use of human volunteers were followed during this research and that the IDEFICS study passed the Ethics Review process of the Sixth Framework Programme (FP6) of the European Commission. Ethical approval was obtained from the relevant local or national ethics committees by each of the eight study centers: the Ethics Committee of the University Hospital Ghent (Belgium); the National Bioethics Committee of Cyprus (Cyprus); the Tallinn Medical Research Ethics Committee of the National Institutes for Health Development (Estonia); the Ethics Committee of the University of Bremen (Germany); the Scientific and Research Ethics Committee of the Medical Research Council, Budapest (Hungary); the Ethics Committee of the Health Office, Avellino (Italy); the Ethics Committee for Clinical Research Aragon (Spain); and the Regional Ethical Review Board of Gothenburg (Sweden). All parents of the participating children gave written informed consent to data collection, examinations, collection of samples, subsequent analysis and storage of personal data and collected samples. Additionally, each child gave verbal consent after being verbally informed in simple terms, due their young age, about the study by a study nurse. This consent process was not further documented, but it was subject to central and local training and quality control procedures. Study participants and their parents could consent to single components of the study, while abstaining from others. All procedures were approved by the above-mentioned Ethics Committees.

## 3. Results

The study population at baseline is shown in Table 1. This study has an almost equal distribution of boys and girls in all countries and a mean age of 6.1 years at baseline. There was large inter-country variability in the proportion of children with a high intake of carbohydrate from starch and sugar respectively: generally low in Spain and high in Hungary and Italy. Short sleep duration also varied by survey country, with the lowest proportion of children categorized as short sleepers in Belgium (2.5%) and the highest in Estonia (69.6%).

Subjects who reportedly consumed breakfast consumed more morning sugar (33 g *vs.* 25 g), morning starch (26 g *vs.* 20 g), midday starch (56 g *vs.* 52 g) and evening starch (28 g *vs.* 26 g) than those reporting no breakfast. Breakfast-eaters reported consuming less midday sugar (34 g *vs.* 37 g) and less evening sugar (22 g *vs.* 29 g) than those reporting no breakfast (results not shown in tables).

In Table 2, we show cross-sectional differences in a range of baseline variables by sleep duration, adjusted for age and country at baseline. Short sleepers had significantly higher BMI z-scores than those sleeping 10 h or more. Raw numbers indicated that short sleep duration was associated with lower probability of high starch intake in the morning but the association was reversed when adjusted for age and survey country, as indicated by a positive beta-coefficient. Furthermore, regression analyses showed that short sleep was associated with less sugar intake at midday but more sugar intake in the evening. The cross-sectional analysis conducted at baseline was repeated cross-sectionally at follow-up without notable difference and is therefore not shown.

Table 3 shows mutually adjusted multivariable linear regression of BMI z-scores at baseline as a function of exposure to high starch and sugar intake at three time-points (morning, midday, and evening) and short sleep duration (referent group “normal to long sleep”), with adjustment for age, sex, high parental education level and survey country. At baseline, a significant positive association was observed between higher BMI z-scores and short sleep duration, HC intake from morning starch and higher values of total EI. A significant inverse association with BMI z-scores was observed for eating breakfast, high parental education level and HC evening starch.

When it came to prediction of two-year BMI z-scores, protective associations with both breakfast consumption and high household parental education level were found. Characteristics associated with increased BMI z-scores included high intake of carbohydrate from morning starch and high intake of overall energy in a model mutually adjusted for age, sex, parental education, survey country and intervention *vs.* control study region but unadjusted for baseline BMI z-scores. When baseline BMI z-scores were included in the model, only high parental education was inversely associated with increasing BMI z-scores. HC from starch in the morning approaches significance (Regression coefficient = 0.05, *p* = 0.060; 95% CI = −0.001; 0.095).

**Table 1:** Characteristics of the study sample and distribution of covariates by country at baseline 2007/2008.

Variable	Belgium	Cyprus	Estonia	Germany	Hungary	Italy	Spain	Sweden	All
	*n* = 277	*n* = 699	*n* = 621	*n* = 1181	*n* = 519	*n* = 1367	*n* = 411	*n* = 869	*n* = 5944
Intervention area, *n* (%) *vs.* control	164 (59.2)	268 (38.3)	241 (38.8)	652 (55.2)	272 (52.4)	794 (58.1)	368 (89.5)	394 (45.3)	3153 (53.1)
Mean age (SD)	5.5 (1.6)	6.2 (1.4)	6.5 (1.9)	6.1 (1.8)	6.5 (1.7)	6.1 (1.8)	5.5 (1.9)	5.9 (2.0)	6.1 (1.8)
Boys, *n* (%)	150 (54.2)	350 (50.1)	292 (47.0)	603 (51.1)	265 (51.1)	708 (51.8)	220 (53.5)	451 (51.9)	3039 (51.1)
Overweight, including obese, *n* (%), Cole 2012	17 (6.1)	152 (21.8)	102 (16.4)	170 (14.4)	75 (14.5)	543 (39.7)	75 (18.3)	77 (8.9)	1211 (20.4)
Mean BMI-z score at baseline (SD), Cole 2012	−0.09 (0.9)	0.40 (1.2)	0.24 (1.1)	0.19 (1.1)	0.11 (1.1)	0.97 (1.2)	0.37 (1.0)	0.08 (0.9)	0.37 (1.2)
Short sleep (<10 h/night), *n* (%)	7 (2.5)	272 (38.9)	432 (69.6)	76 (6.4)	219 (42.2)	815 (59.6)	128 (31.1)	112 (12.9)	2061 (34.7)
High parental education, *n* (%)	134 (48.9)	319 (52.7)	83 (13.7)	186 (16.5)	263 (51.1)	258 (19.0)	226 (55.5)	593 (69.5)	2062 (35.9)
Breakfast on weekdays, *n* (%)	271 (98.2)	667 (95.4)	483 (81.3)	913 (77.3)	234 (46.6)	1221 (89.6)	317 (77.1)	705 (94.5)	4811 (83.4)
Energy kcal/day on weekdays, mean (SD)	1383 (366)	1421 (362)	1718 (442)	1524 (439)	1517 (442)	1697 (404)	1540 (385)	1545 (401)	1569 (424)
Mean sugar (g), morning (SD)	39 (22)	23(13)	23 (18)	48 (32)	33 (30)	26 (17)	34 (17)	28 (17)	32 (24)
Mean starch (g), morning (SD)	25 (15)	38 (23)	17 (15)	28 (17)	27 (27)	19 (15)	18 (13)	26 (16)	25 (19)
Mean sugar (g), midday (SD)	33 (23)	20 (15)	49 (30)	43 (30)	31 (29)	34 (22)	36 (20)	30 (19)	35 (26)
Mean starch (g), midday (SD)	35 (18)	39 (23)	46 (27)	32 (26)	51 (33)	101 (55)	41 (20)	47 (23)	55 (43)
Mean sugar (g), evening (SD)	23 (19)	12 (12)	39 (26)	24 (22)	37 (26)	17 (16)	20 (14)	26 (19)	24 (21)
Mean starch (g), evening (SD)	27 (19)	25 (20)	28 (20)	22 (16)	23 (19)	37 (36)	12 (12)	36 (19)	28 (25)
HC sugar, morning, *n* (%)	99 (36)	55 (8)	73 (11)	596 (50)	178 (34)	239 (17)	110 (27)	135 (16)	1485 (25)
HC starch, morning, *n* (%)	60 (22)	334 (48)	77 (12)	403 (34)	172 (33)	186 (14)	42 (10)	212 (24)	1486 (25)
HC sugar midday, *n* (%)	64 (23)	43 (6)	271 (44)	446 (38)	108 (21)	314 (23)	104 (25)	136 (15)	1486 (25)
HC starch midday, *n* (%)	18 (7)	67 (10)	100 (16)	69 (6)	136 (26)	917 (67)	31 (8)	148 (17)	1486 (25)
HC sugar evening, *n* (%)	79 (29)	42 (6)	322 (52)	307 (26)	261 (50)	193 (14)	363 (48)	234 (27)	1486 (25)
HC starch, evening, *n* (%)	55 (20)	163 (23)	157 (25)	152 (13)	83 (16)	495 (36)	20 (5)	361 (42)	1486 (25)

Footnote: household parental education level was categorized according to the International Standard Classification of Education (ISCED) and the original six ISCED levels [31] were then combined into two levels (not high *versus* high). ISCED levels 0–4 constitute “not high” education while levels 5 and 6 are defined as high education. Breakfast on weekdays was categorized as yes or no, based on the weekday 24-h recalls included in this study. High carbohydrate (HC-starch and HC-sugar categories) were calculated for starch and sugar at three time-points (morning, midday and evening) by assigning those in the highest quartile for grams of sugar/total energy intake (EI) and grams of starch/total EI to the HC groups. The number and percentage of children in each HC sub-category are given in the lower half of the table.

**Table 2:** Cross-sectional baseline characteristics of short sleepers (<10 h). The right-hand columns present the regression coefficients, with 95% CI (Confidence Interval).

Outcome	Not Short Sleeper *n* = 3883	Short Sleeper *n* = 2061	Regression Coefficient	95% CI
BMI score, Cole 2012	0.25	0.61	0.12	0.05; −0.18
**Dietary factors modeled in multivariable model shown in Table 3**				
HC morning sugar, n (%)	1080 (27.8)	405 (19.7)	0.03	−0.13; 0.19
HC morning starch, n (%)	997 (25.7)	489 (23)	0.21	0.05; 0.37
HC midday sugar, n (%)	987 (25)	499 (24)	−0.16	−0.31; −0.00
HC midday starch, n (%)	754 (19)	732 (36)	−0.08	−0.24; 0.08
HC evening sugar, n (%)	916 (24)	570 (28)	0.21	0.05; 0.36
HC evening starch, n (%)	887 (23)	599 (29)	0.07	−0.08; 0.22
Energy, kcal/day, mean	1522.8	1655.6	31.74	7.69; 55.79
Eats breakfast, %	84.17	81.82	−0.03	−0.047; −0.00
**Other factor modeled in multivariable model shown in Table 3**				
High parent education %	39.5	29.1	−0.029	−0.06; 0.00
**Descriptive dietary factors, not in multivariable model shown in Table 3**				
Starch, g/day	99.8	122.4	3.92	1.29; 6.55
Sugar, g/day	91.8	87.9	0.52	−1.88; 2.92
Carbohydrate, energy-%, day	52.3%	52.2%	0.01	−0.64; 0.65
Fat, energy-%, day	31.5%	31.0%	0.09	−0.43; 0.61
Protein, energy-%, day	15.8%	16.0%	−0.29	−0.55; 0.03

Footnote: High carbohydrate (HC-starch and HC-sugar categories) were calculated for starch and sugar at three time-points (morning, midday and evening) by assigning those in the highest quartile for grams of sugar/total energy intake (EI) and grams of starch/total EI to the HC groups.

**Table 3:** High carbohydrate intake from sugar and starch at three times of day and short sleep duration and association with BMI z-scores at baseline, adjusted for age, sex and country.

Exposures Measured at Baseline (*n* = 5750) ^†^	Regression Coefficient	95% CI
Short sleep < 10 h *versu*s ≥ 10 h	0.09	0.02; 0.16
Breakfast (yes v. no)	−0.28	−0.36; 0.19
HC morning sugar	−0.02	−0.09; 0.05
HC morning starch	0.12	0.05; 0.20
HC midday sugar	0.03	−0.04; 0.10
HC midday starch	0.02	−0.07; 0.10
HC evening sugar	−0.04	−0.12; 0.03
HC evening starch	−0.08	−0.15; −0.00
Total energy intake in kcal	0.03	0.02; 0.04
Parental education (high v. “not high”)	−0.09	−0.16; 0.24

**^†^** *n* reduced from 5944 at baseline due to non-response on two variables: breakfast (*n* = 5772) and Parental Education (*n* = 5750). High carbohydrate (HC-starch and HC-sugar categories) were calculated for starch and sugar at three time-points (morning, midday and evening) by assigning those in the highest quartile for grams of sugar/total energy intake (EI) and grams of starch/total EI to the HC groups. CI: Confidence Interval.

**Figure 3:**
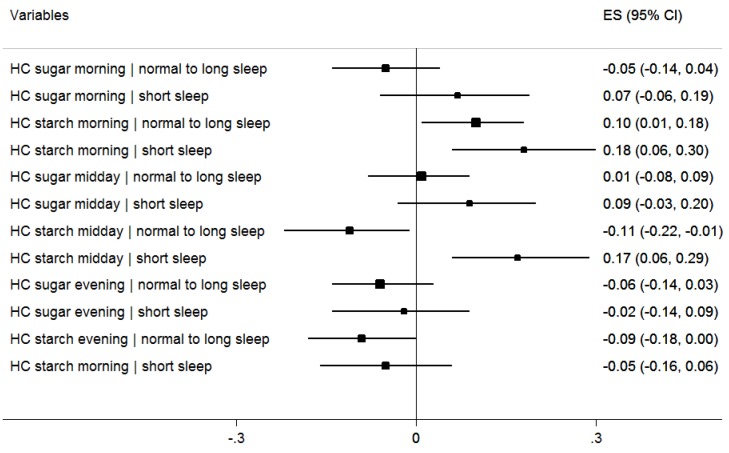
Effect of HC intake (sugar and starch) at 3 meal occasions on BMI z-scores at baseline by sleep duration. ES = effect size (regression coefficient). *n* reduced from 5944 at baseline due to non-response on two variables: breakfast and parental education level (*n* = 5750); Linear regression of BMI z-scores on sleep duration, HC intake variables, and their respective product terms, further adjusted for eating breakfast, parental education, age, sex, and survey country. Long sleep represents normal and long sleep ≥10 hours. CI: Confidence Interval.

In Figure 3, the effect of short sleep duration on the relationship between HC from sugar and starch at each meal occasion and BMI z-scores is shown in a mutually adjusted model including age, sex, parental education, total EI, eating breakfast and survey country. Six interaction terms were introduced to the main effects, one of which was significant, *i.e.*, the interaction between high starch at midday and short sleep. In children with short sleep duration, the risk posed by HC intake from starch at midday had a stronger effect in those with short sleep duration. We also observed that HC from starch in the evening among the children without short nocturnal sleep duration is inversely associated with BMI z-scores but the respective interaction term was not significant.

There was a positive interaction between high midday sugar and short sleep duration with respect to BMI z-scores at two-year follow-up, but it was no longer observed after adjustment for baseline BMI z-scores (not shown). After adjustment for baseline BMI z-scores, only high parental education level remained significantly protective, (Regression coefficient = −0.08, *p* = 0.001, 95% CI = −0.12; −0.03; results not shown in tables).

## 4. Discussion

Our findings demonstrate that a high proportion of carbohydrate from starch in the morning is associated with an increased risk of a higher BMI z-scores at baseline in children participating in the IDEFICS study. Moreover, we found that high intake from starch at midday, in combination with short sleep duration, poses a risk for overweight. We also showed that eating breakfast is associated with lower BMI z-scores cross-sectionally at baseline, concurring with previous research [13,16] However, at the cross-sectional two-year follow-up analysis, eating breakfast offered no protection from increasing BMI z-scores, as has also been reported [17,18].

There is limited research on macronutrient distribution in children’s diets. However, a systematic review of intervention studies published in 2014 examined the impact of dietary macronutrients on BMI and cardio-metabolic outcomes in overweight and obese children and adolescents [32]. From 14 eligible studies, five examined low carbohydrate intake related to BMI [32]. A meta-analysis of these low-carbohydrate studies in children and adolescents indicated a greater reduction in BMI in the low-carbohydrate group immediately after dietary intervention, but the authors noted that the quality of the studies was limited [32]. While our study was observational, our findings are in line with those of the systematic review of intervention studies, as we observed an association between HC intake from starch and higher BMI z-scores.

In adults, de Castro has reported that the time of day is an important factor influencing overall intake [33]. In the same cohort of adults, de Castro demonstrated that when the proportion of daily carbohydrate ingested in the morning was high, less total food energy and carbohydrate were consumed over the entire day [34]. In contrast, we found that high morning starch intake is associated with higher BMI z-scores and that breakfast-eaters consumed more total energy than those not reporting breakfast, which indicates that those skipping breakfast do not make up for skipped breakfast energy later in the day. Although de Castro did not report specifically on midday HC intake or on associations with overweight, his work may help to explain our finding that high starch intake at midday, interacting with short sleep duration, was associated with higher BMI z-scores, compared with morning carbohydrate intake. A study conducted on adults consuming a low-calorie diet studied the effects of carbohydrates consumed mostly at dinner and reported that subjects on the experimental diet underwent positive hormonal changes (leptin and adiponectin concentrations) and reported less hunger [35]. Similarly, we observed that high starch intake in the evening was inversely associated with BMI z-scores cross-sectionally at baseline. Sofer *et al.* [35] reported that simple dietary manipulation of carbohydrate distribution appears to yield additional benefits beyond calorie reduction. The hormone leptin regulates hunger, satiety and EI. Previous studies have reported that leptin secretion falls between 8:00 a.m. and 4:00 p.m., reaching the lowest point at 1:00 p.m., increasing at 4:00 p.m. and typically peaking at 1:00 p.m. [36,37]. This indicates that leptin levels are lowest at midday, which may be related to our findings that HC intake from starch in the morning and HC intake from starch at midday, interacting with short sleep duration, are positively associated with higher BMI z-scores.

It may be of particular interest that our cross-sectional findings are attributable to starch rather than sugar intake; there are plausible explanations for this. Most importantly, we report on all sugars, rather than added sugars, as a component of carbohydrate. The United States Department of Agriculture (USDA) recommends that children consume the following daily: two cups or approximately 350 g of fruit, 2.5 cups or approximately 475 g of vegetables, 6 oz. or approximately 168 g of whole grains and three cups or approximately 720 mL of dairy products. Following the USDA recommendations, a child exceeds 80 g of sugar intake when consuming just 1530 kcal per day [38]. The mean sugar intake in our study sample was 90.5 g, while the mean EI was 1569 kcal. This might indicate that added sugars do not contribute significantly to the dietary intake in our study population, *i.e.*, the sugars consumed occur naturally in foods considered to be healthy. However, it is also possible that added sugars or “bad” sugars may be under-reported by parents and therefore deflate the estimate of overall sugar intake. While we have eliminated energy over- and under-reporters from our sample based on total caloric intake, we cannot exclude the existence of a “good” food–“bad” food reporting bias.

Similar to our sleep findings, previous research has demonstrated a relationship between adiposity and short sleep duration [39,40]. Recently, a Belgian study confirmed that short sleep duration is associated with central adiposity in children [7]. We found that those with short sleep duration (<10 h) had higher BMI z-scores than those with 10 or more hours of sleep. Previous research has demonstrated that acute sleep deprivation increases snack portions [41] and that lack of sleep is associated with increased snacking, an increased number of daily meals and a preference for energy-rich foods [42]. The previously-mentioned study of Malaysian children demonstrated that children who sleep less than the recommended number of hours consumed more carbohydrates and were at higher risk of overweight/obesity [10]. Our findings indicate that children with shorter sleep duration are more at risk of overweight and that inadequate sleep potentiates the association between increased BMI z-scores and HC intake. It may be speculated that a HC diet combined with inadequate sleep may represent part of the explanation for high rates of obesity in children from a lower socioeconomic position (SEP). A number of previous studies, including the IDEFICS study, have reported that children from higher-income or more educated families tend to eat more healthfully [43,44,45,46,47]. However, in the context of the associations reported here, it should be pointed out that our findings are statistically independent of SEP, as defined by household parental education level.

Our findings on breakfast may be of interest, in light of inconsistencies in the literature. Our cross-sectional results at baseline are in line with other European studies reporting an association between breakfast intake and lower BMI, compared with children who skip breakfast [13,14,15,16]. However, our findings at two-year follow-up show no consistent association between breakfast consumption and lower BMI z-scores support those of a 2010 systematic review that concluded causality has not been demonstrated [17]. Similarly, a study of Danish children aged two and five also found no association between breakfast consumption and overweight [48]. Furthermore, it might be surprising that children with short sleep duration were less likely to eat breakfast than those who slept 10 or more hours, as it might be expected that those with more waking hours would have more time to consume breakfast. While the difference was statistically significant (82%: <10 h *versus* 84%: ≥10 h), the absolute values were of low magnitude.

To our knowledge, this is the first study to simultaneously examine carbohydrate intake and its temporal distribution, short nocturnal sleep duration, breakfast habits and risk of overweight in a large cohort of children with diverse dietary cultures and lifestyle habits. Moreover, this study adhered to strict implementation of standardized operating procedures during fieldwork as well as plausibility checks during data entry. However, our study is not without limitations, the most important of which is including only one day of dietary recall. It is also important to note that the calculations are heterogeneous because macronutrients are calculated from a number of different FTC. For example, in Sweden, Germany and Hungary sugar was calculated as monosaccharide plus disaccharide, while in Estonia sugar consists of glucose, fructose, sucrose, maltose and lactose. These discrepancies might lead to subtle difficulties in comparing dietary carbohydrate intake. Finally, it should be kept in mind that the sample included in the IDEFICS study was not randomly selected and the related descriptive data cannot be considered to be representative at the country level.

## 5. Conclusions

This study showed BMI z-scores were cross-sectionally associated with high dietary carbohydrate intake from starch consumed in the morning, and in children with less than optimal sleep duration with high dietary carbohydrate intake from starch at midday. Breakfast consumption was not consistently associated with BMI z-scores. These findings suggest that childhood overweight might be reduced by limiting the proportion of dietary carbohydrates, particularly from starchy foods, in morning meals, and even more so in midday meals, and by encouraging the recommended amount of sleep each night.
